# *In vitro* and in *silico* multidimensional modeling of oncolytic tumor virotherapy dynamics

**DOI:** 10.1371/journal.pcbi.1006773

**Published:** 2019-03-05

**Authors:** David R. Berg, Chetan P. Offord, Iris Kemler, Matthew K. Ennis, Lawrence Chang, George Paulik, Zeljko Bajzer, Claudia Neuhauser, David Dingli

**Affiliations:** 1 Department of Information Technology, Mayo Clinic, Rochester, Minnesota; 2 Molecular Medicine, Mayo Clinic, Rochester, Minnesota; 3 Boston Children’s Hospital and Boston Medical Center, Boston, Massachusetts; 4 International Business Machines, Rochester, Minnesota; 5 Department of Biochemistry and Molecular Biology, Mayo Clinic, Rochester, Minnesota; 6 Department of Mathematics, University of Houston, Houston, Texas; University of California Irvine, UNITED STATES

## Abstract

Tumor therapy with replication competent viruses is an exciting approach to cancer eradication where viruses are engineered to specifically infect, replicate, spread and kill tumor cells. The outcome of tumor virotherapy is complex due to the variable interactions between the cancer cell and virus populations as well as the immune response. Oncolytic viruses are highly efficient in killing tumor cells in vitro, especially in a 2D monolayer of tumor cells, their efficiency is significantly lower in a 3D environment, both in vitro and in vivo. This indicates that the spatial dimension may have a major influence on the dynamics of virus spread. We study the dynamic behavior of a spatially explicit computational model of tumor and virus interactions using a combination of in vitro 2D and 3D experimental studies to inform the models. We determine the number of nearest neighbor tumor cells in 2D (median = 6) and 3D tumor spheroids (median = 16) and how this influences virus spread and the outcome of therapy. The parameter range leading to tumor eradication is small and even harder to achieve in 3D. The lower efficiency in 3D exists despite the presence of many more adjacent cells in the 3D environment that results in a shorter time to reach equilibrium. The mean field mathematical models generally used to describe tumor virotherapy appear to provide an overoptimistic view of the outcomes of therapy. Three dimensional space provides a significant barrier to efficient and complete virus spread within tumors and needs to be explicitly taken into account for virus optimization to achieve the desired outcome of therapy.

## Author summary

Tumor therapy with replicating oncolytic viruses is based on the premise that if the tumor specific virus infects and is amplified by the tumor population and spreads sufficiently within the tumor, it will lead to eradication of the cancer. The outcome of this approach is an exercise in population dynamics, and, as in ecology, depends on the detailed interactions between the various players involved. Mathematical models have been used to capture these dynamics, but space is often explicitly excluded from these models. We combine in vitro experiments studying tumor growth and virus spread in two and three dimensions to inform the development of a spatially explicit computational model of tumor virotherapy and compare the outcome with non-spatial, mean-field models. Viruses generally spread from cell to cell, and therefore the number of nearest neighbors close to an infected cell is important. Experimental data show that in three dimensions, the median number of nearest neighbors is 16 compared to 6 in the 2D plane. However, while simulations in 3D reach equilibrium faster than in 2D, tumor eradication is much less common in 3D than in 2D. Three dimensional space plays a critical role in the outcome of tumor virotherapy and this additional spatial dimension cannot be ignored in modeling.

## Introduction

Tumor therapy with replication competent viruses (oncolytic virotherapy) is an exciting new field of therapeutics. In principle, amplification of the virus in target cancer cells could allow ongoing spread of the infection within the tumor and its eventual elimination [1, 2]. The advantages of recombinant viruses for cancer therapy include (i) specific engineering for infection, replication and killing of tumor cells [1], (ii) amplification of the therapy itself by the tumor, (iii) stimulation of an anti-tumor immune response by breakdown of tumor immune tolerance [3], (iv) a bystander effect especially if the virus is armed with specific genes such as the sodium iodide symporter (NIS) [4]. With the exception of cancer therapy with recombinant chimeric antigen receptor (CAR-T) T cells, tumor virotherapy is an exercise in population dynamics in which the interactions between the virus, the tumor and the immune system determine the outcome of therapy [5–13].

Many mathematical models have been developed to describe the outcome of such interactions [5, 6, 8–13]. Most models are based on the Lotka-Volterra approach and assume mass action kinetics with well-mixed populations. As a result, the models are helpful in illustrating general principles but lack important features, in particular the spatial geometry of the cells in a tumor, to be of predictive value if applied to in vivo scenarios. This is a critical deficiency especially if we are to attempt optimization of therapy [9]. Durrett and Levin and many others have addressed the problem of spatial constraints on the interactions between populations in ecological systems [14–16 and reference therein]. More recently, Paiva et al described a three-dimensional computational simulator of tumor and virus interactions and concluded that complex dynamics are in place with the spatial arrangements between cells being important determinants of outcome [17]. Reis et al reported on a 3D computational model of cancer therapy that illustrated the important differences when considering dynamics in 2 versus 3 dimensions and how restricted the parameter space may be to achieve tumor eradication [18]. Wodarz and colleagues have reported on their work with agent based modeling of tumor virotherapy where space is explicitly considered [7, 19]. Using experimental data on the spread of adenovirus in a monolayer (2D) of 293T cells as a guide, they showed that various patterns of virus spread such as ‘hollow ring structure’, ‘filled ring structure’ and a ‘dispersed pattern’ are possible and how space and virus/tumor cell parameters can interact to determine the outcome of therapy [7]. Ring structure formation is associated with a quadratic growth of the virus population which subsequently becomes linear. The dispersed pattern of spread is invariably associated with therapeutic failure while the ring structures may be associated with a cure especially if the center of the ring is associated with elimination of the target population and the virus continues to expand radially and catch up with all the target population (since a boundary will be reached). Wodarz and colleagues found that the local dynamics on a smaller scale can predict the outcome of the spatially explicit system [7]. Interestingly, the experiments also showed two patterns of infection–limited spread versus robust expansion of the infected cell population. Which pattern the infection followed was established early on. Subsequently, they showed that in part this dichotomy in outcomes was due to interferon induction in infected cells that inhibited virus spread [19]. This suggests that there is a local race between spread of the virus against the development of an interferon response which limits viral replication. Modeling suggests that multiple infections by the virus are also necessary to explain the dynamics, especially when the populations are small.

However, many modeling approaches described to date have generally lacked any experimental data to validate them. To address this problem, we have developed an *in silico* computational model that captures the dynamics between the tumor and virus populations in a spatially explicit manner (two and three dimensions). We use in vitro 2D and 3D data to inform the model parameters and then use the computational model to explore various critical properties of oncolytic viruses. We show that the introduction of a third dimension alters the dynamics significantly and that this has important implications for the outcome of therapy.

## Results

### In vitro cell dynamics

**Fig 1:**
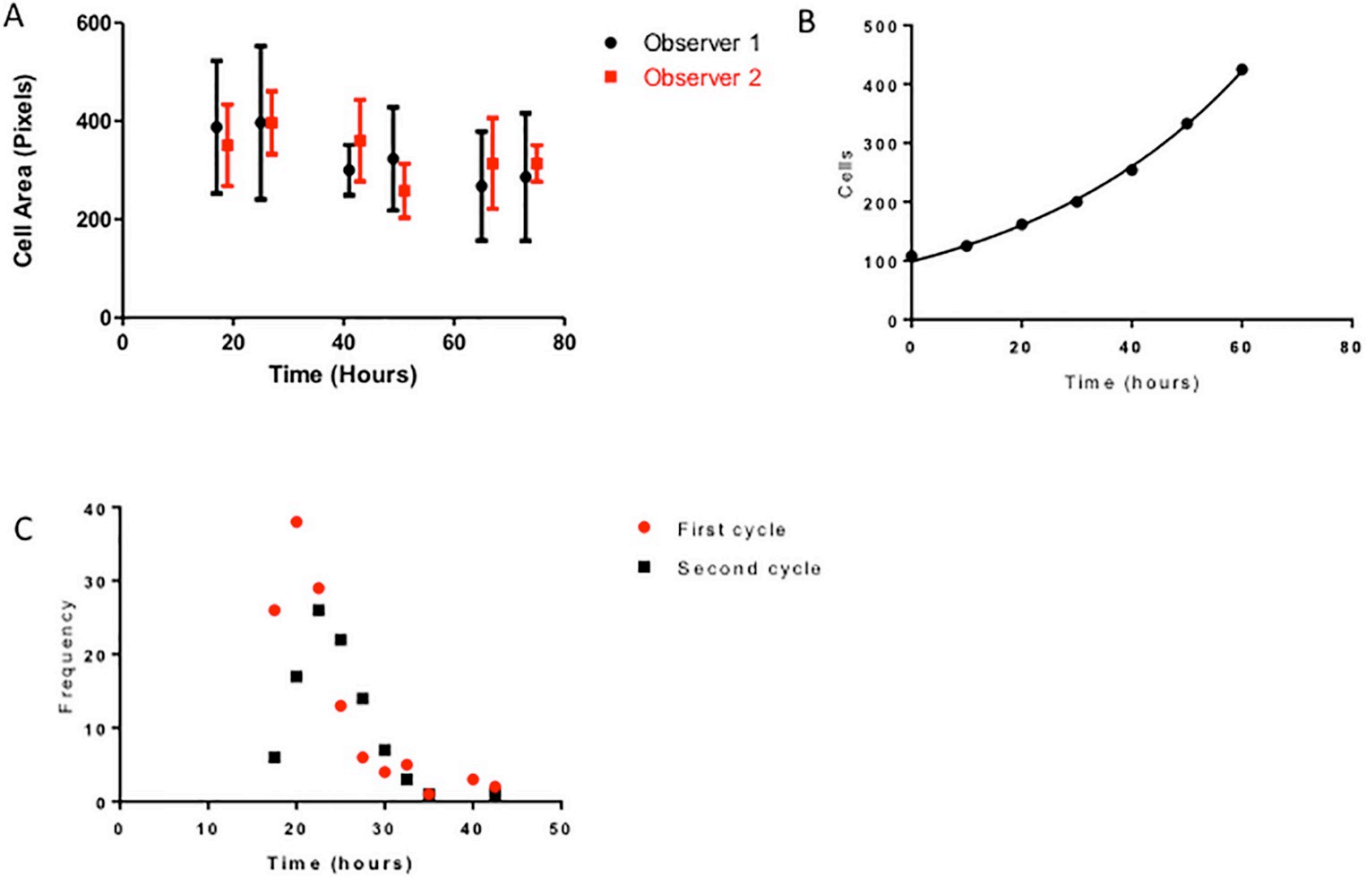
In vitro cell kinetics. (A) Two independent observers measured the pixel area of cells growing in culture at different time points and compared them to the phase contrast images. There was excellent agreement between the two observers. (B) Tumor cells grow exponentially in culture (*R*^2^ = 0.98, *p* < 0.01). (C) Distribution of replication rates for individual cells in culture.

To quantitate cell populations based on fluorescent imaging, we initially determined the pixel area that represents a cell. Two independent observers quantitated the number of cells (n = 375 cells per time point per observer) in a given area based on phase contrast images and the pixel area of the cells in the corresponding fluorescent images. The median number of pixels was 326.9 versus 323 (p = 0.8723, Mann Whitney) for each observer. There was excellent agreement between the two observers (Fig 1A). This means that the inter observer variability in cell area was 1.1%. The number of cells present in a growing population was measured at 6 different time points. We determined that the cell populations grew exponentially in 2D culture (Fig 1B). The estimated doubling time for the population was 28 hours based on an exponential fit to the data. In contrast, the rate of replication of *individual* cells based on serial tracking was estimated to be 21.9 hours (Fig 1C) and varied from 20 hours for the first replication (n = 127 events) to 22.5 hours for the second (n = 97 events). The difference between these two observations was not statistically significant (Wilcoxon sign rank test, p = 0.6563). There was a strong positive correlation between the two cell cycle times observed (Spearman’s rho = 0.795, p = 0.0072).

**Fig 2:**
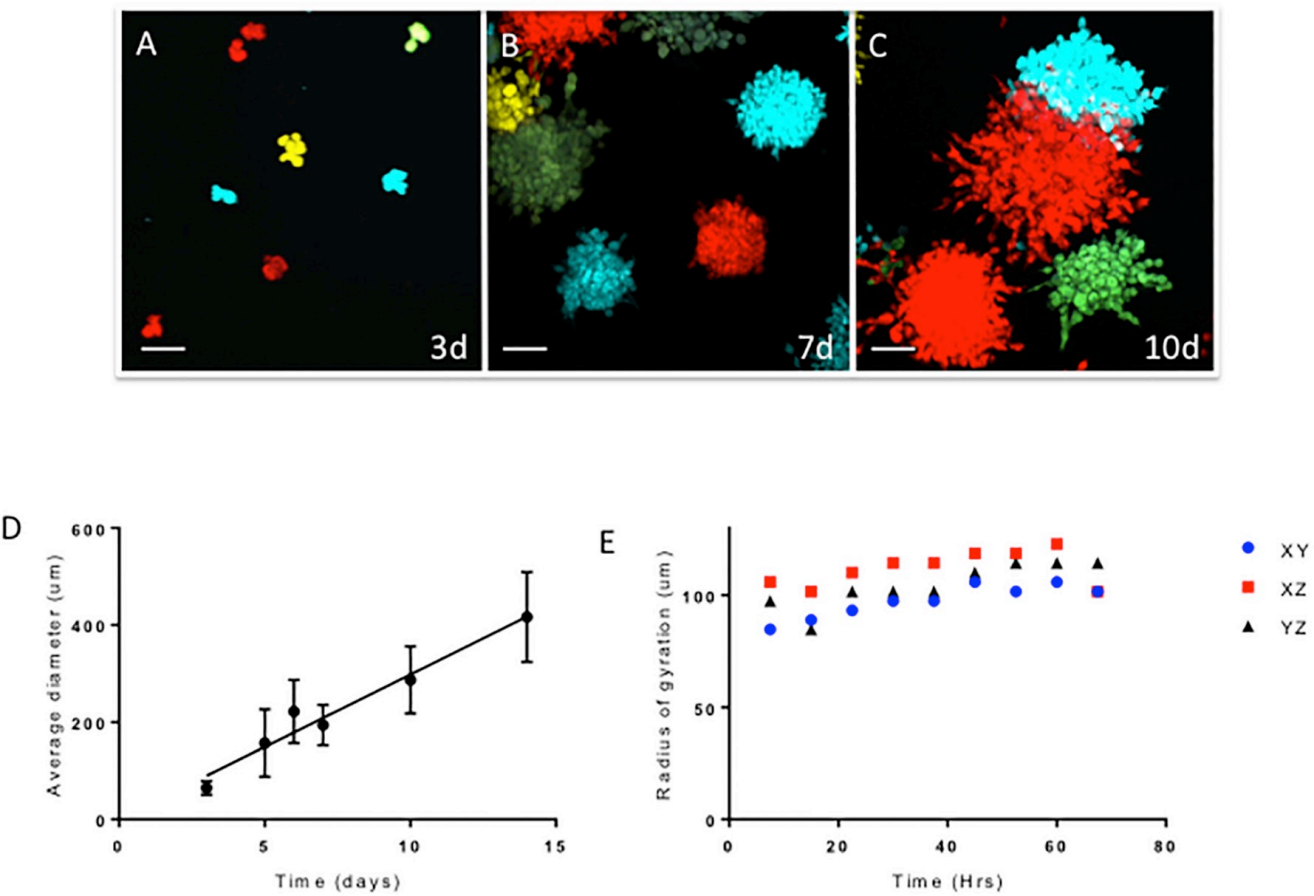
Tumor spheroid growth in culture. (A) HT1080 cells (5000 cells) engineered to express fluorescent proteins were grown in culture under conditions to promote spheroid formation. Maximum intensity projections of cells imaged 3 days (A), 7 days (B) or 10 days (C) after plating are shown. Each spheroid is monochromatic, implying that each arose from a single cell. Scale bar = 100μm. (D) Spheroid growth is linear in time and the spheroids maintained their symmetric shape as measured by the radius of gyration (E).

We measured the growth of tumor cells in 3 dimensions serially by imaging multiple spheroids at specific time points (Fig 2A). Each spheroid was monochromatic, implying that each spheroid arose from one founder cell even though a mixture of HT1080 cells with all 4 colors (blue, yellow, green and red) were plated. We found a linear increase in diameter of the spheroids as a function of time although there was considerable variability as the spheroids grew (Fig 2D). The median increase in spheroid diameter was 15μm/day or 0.63 μm/hr. In addition, we also determined the radius of gyration of representative spheroids (*n* = 12) across the 3 axes of growth as a function of time [20]. As can be seen from Fig 2E, the tumor cells growing in 3D generally retained a spherical shape with a median radius of gyration of 97.5μm in the XY plane, 114μm in the XZ plane and 101.7 μm in the YZ plane. Given that the average diameter of a cell is ≈10μm, our observations suggest that the variability in the radius of gyration was of approximately 1 cell in any axis and therefore growth of the spheroids was generally uniform in all directions.

### Nearest neighbors in 2D and 3D

**Fig 3:**
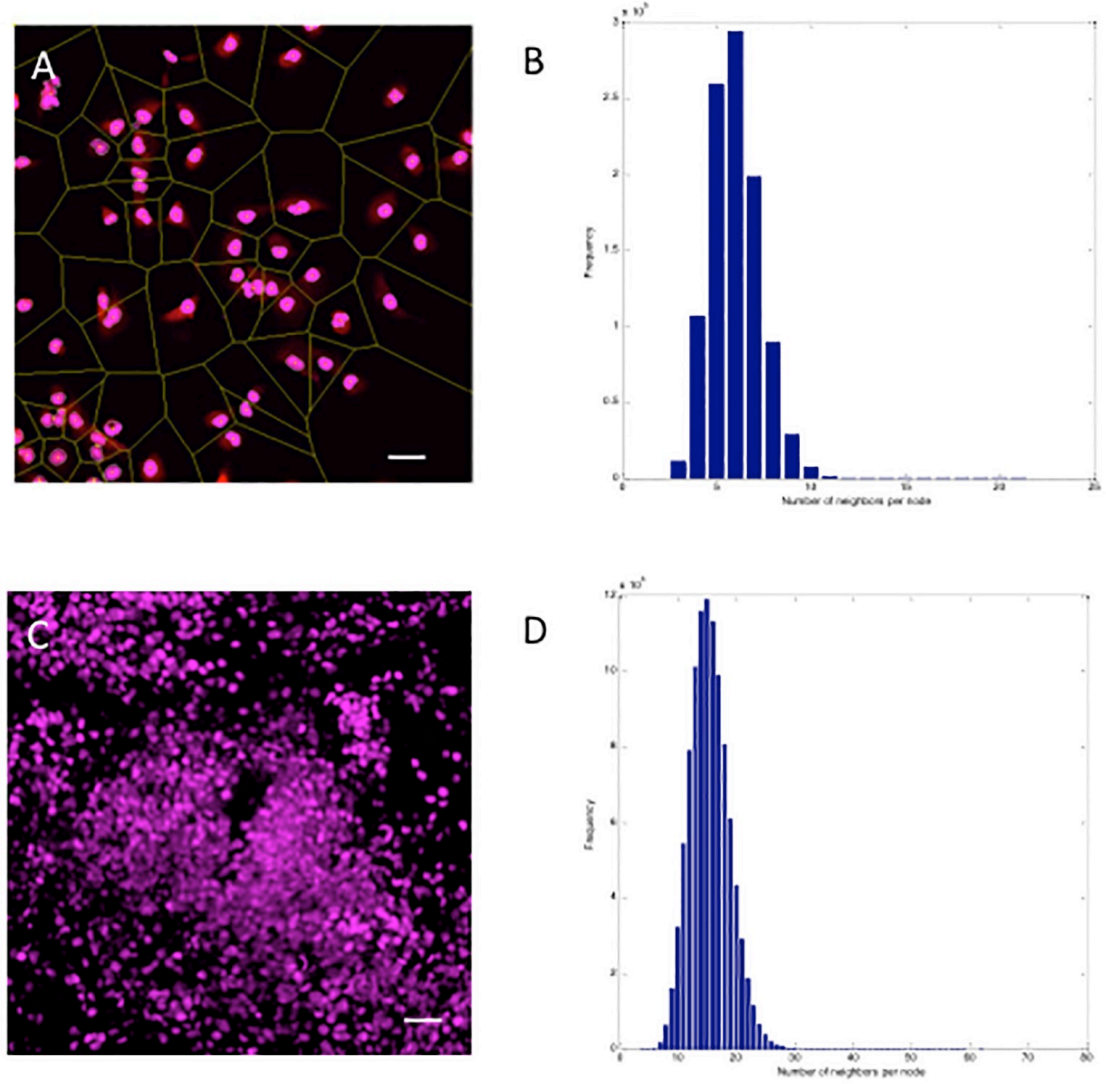
Distribution of nearest neighbors in 2D and 3D culture. The Voronoi tessellation method was used to determine the distribution of nearest neighbors both in 2D (A) and in 3D (C) cultures (using Z stacking). In (C) a maximum intensity projection is shown of a HT1080-EBFP-NLS tumor grown in and excised from a mouse. Scale bar = 50μm. (B) The median number of nearest neighbors in 2D was 6, while this was 16 in 3D culture (D).

Since oncolytic measles viruses (as well as other viruses) generally spread from cell to cell, we hypothesized that the number of cells surrounding any given cell is of critical importance. Therefore, we wanted to determine the number of nearest cell neighbors based on whether cells are growing in the 2D plane versus in 3 dimensions. This data informed the development of the computational model to realistically simulate the in vitro dynamics. We studied cell populations by Voronoi tessellation analysis to determine the distribution of nearest neighbors for cells growing in the 2D (Fig 3A and 3B) plane as well as in spheroids (Fig 3C and 3D). As expected, the number of nearest neighbors was significantly different in 2D versus 3D with a median of 6 (range: 3–10) versus 16 (range: 4–30) neighbors respectively.

### Virus spread in vitro in 2D and 3D

**Fig 4:**
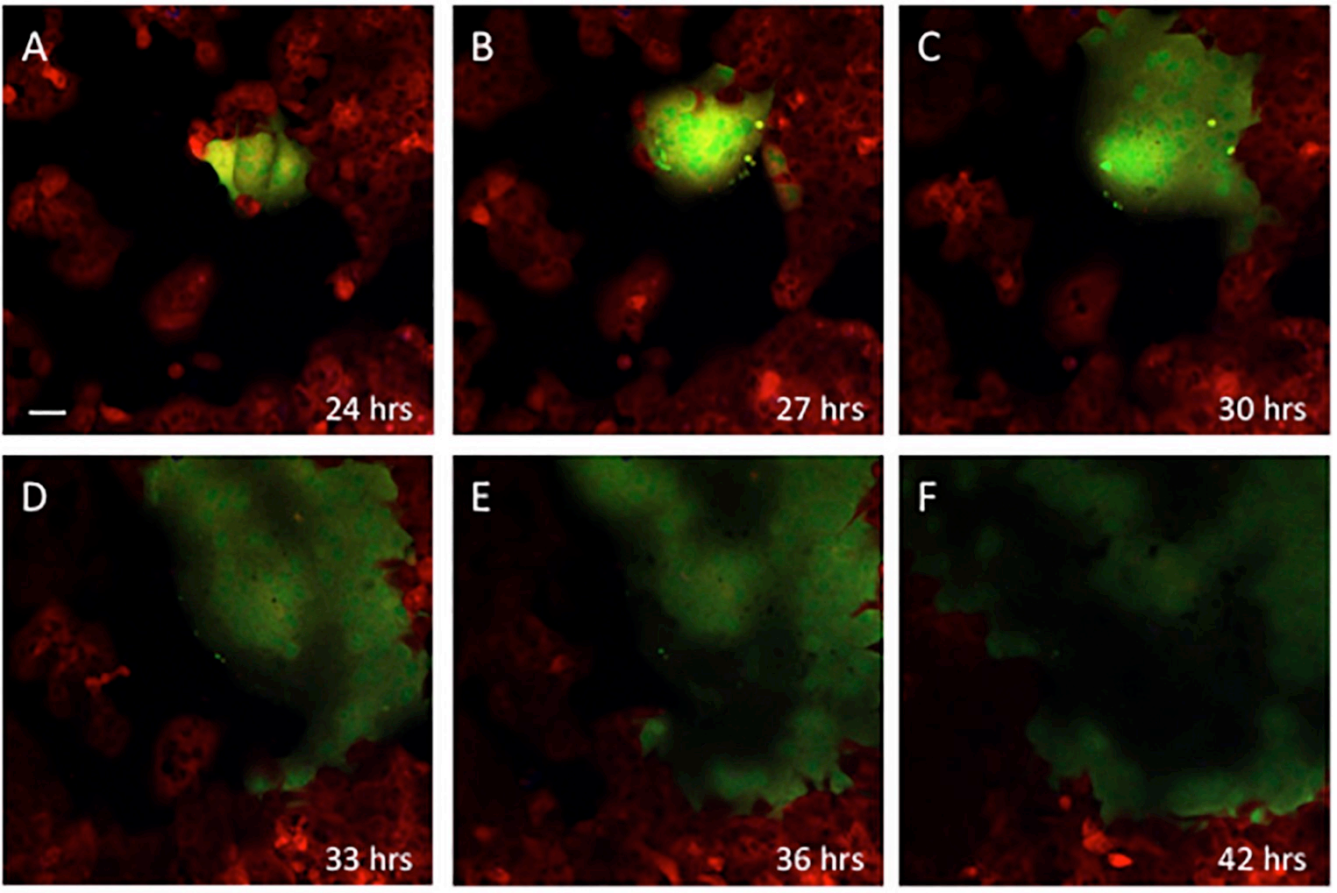
Virus dynamics in 2D. HT1080 tdTomato cells (red) were infected with MVeGFP (MOI = 1) and imaged every 15 minutes starting 24 hours post infection (A). Representative images are shown in (A) to (F). From an initial focus (A), the virus spread by syncytium formation to incorporate the bulk of the tumor population over the course of 42 hours.

**Fig 5:**
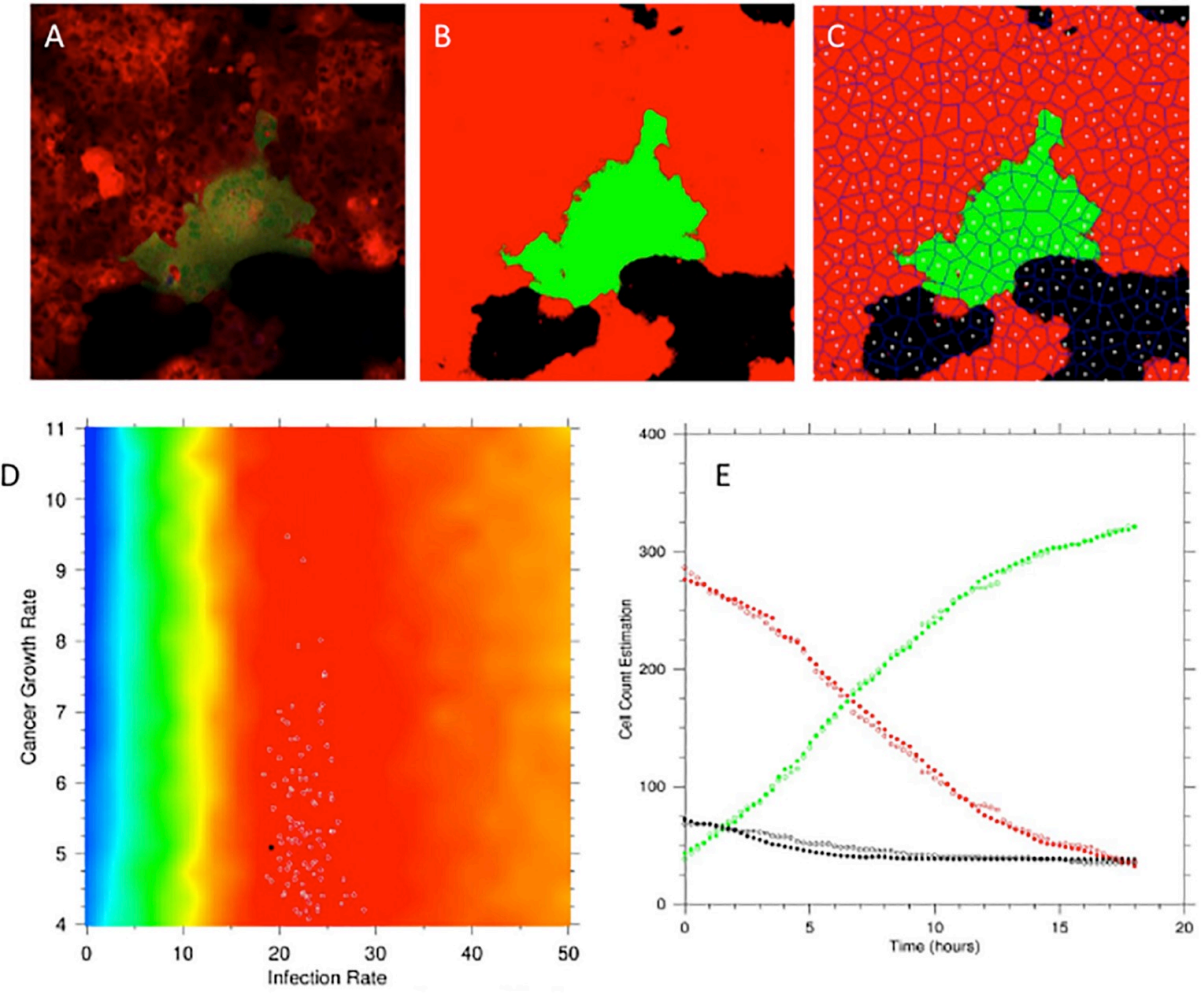
In vitro and in silico correlations (2D). (A) Serial Images of tumor cell and virus spread were captured and converted into digital images (B). Voronoi tessellation (C) and data from the average area of a cell were used to quantitate the populations as a function of time. (D) Fitting of the data to the mean field equations results in a heat map of the goodness of fit–the best result is shown as a black circle. (E) In silico simulations (filled circles) using the best parameter estimates from (D) and the initial conditions capture accurately the actual population dynamics (empty circles). Red represents tumor cells, green represents the infected cell population while black represents the empty space.

We utilized serial imaging studies to determine the rate of growth of the tumor and virus infected cell populations both in the 2D plane and in 3 dimensions. A total of 14 independent experiments were studied in 2D. Fig 4 presents snapshots of the spread of a single focus of infection (green) due to syncytium formation where cells fuse together to form a multicellular object. In Fig 5A–5C, we provide a representative case of data capture, digitalization and then analysis of cell population size by the Voronoi tessellation method (C). Fitting of serial imaging data to the mean field solution (see methods), enabled us to determine the best parameter estimates for cell replication and virus spread (Fig 5D). Although the rates of tumor cell and virus spread varied, the median rate of replication for tumor cells was 4.39 per hour while the virus infection was spreading at a median rate of 18.94 cells/hour which implies that the virus was spreading 4–5 times as quickly as the tumor cell population was growing. A faster rate of spread of the virus compared to tumor cell growth is a necessary condition for any plausible scenario where the virus can eliminate the tumor cell population leading to a potential cure–something that is *consistently* observed in vitro [21, 22] and also predicted by others [7, 19]. We used the *best estimate* of the parameter set obtained from the data fitting (the black dot in Fig 5D) to determine cell population size and compare that to the actual measurements. As can be seen from Fig 5E, the computational output mirrored the experimental results with a high degree of accuracy. In virtually all of our experiments with cells growing in the 2D plane, the virus consistently eliminated the tumor cell population within 48 hours.

**Fig 6:**
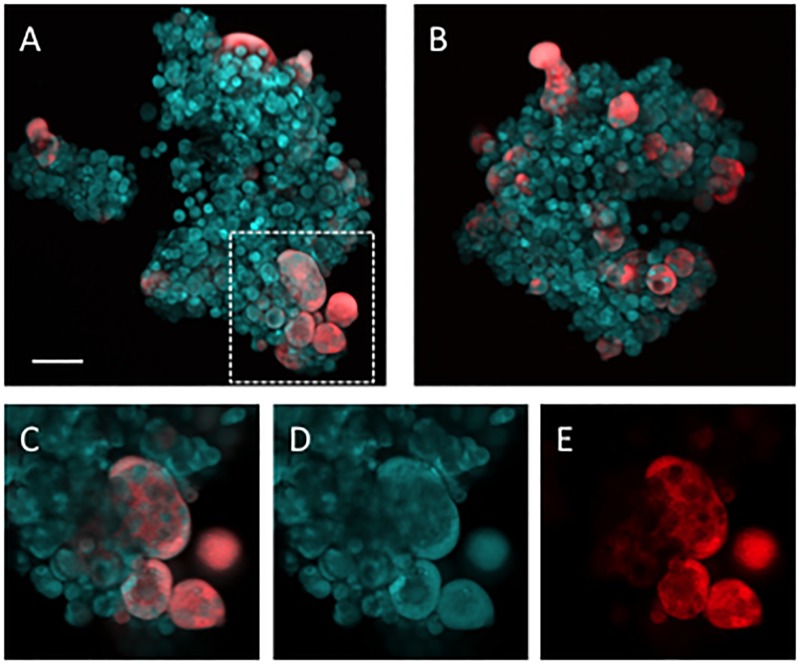
Virus dynamics in tumor spheroids. Representative maximum intensity projections of HT1080-CFP tumor cell spheroids (blue) infected with MV-tdTomato (red) at MOI = 1 (A) or MOI = 10 (B). Scale bar = 100μm. Multiple distinct foci of infection are present with syncytium formation (C–D). Single 2D slice of the area outlined in (A) is enlarged in (C) with individual channels in (D) and (E). However, many cells remain uninfected for the duration of the experiment, unlike the experiments in the 2D environment.

The dynamics of virus spread in 3D tumor spheroids were surprisingly different with the virus spreading more slowly in the 3D environment. Although various independent foci of infection occurred in each spheroid (Fig 6), with the formation of multinucleated syncytia (red), many infected cells remained viable for the duration of the experiment (~7 days). We also observed that many cells in the spheroids never become infected despite being in close proximity to virus-infected cells. More recently we documented syncytium formation in vivo in a mouse dorsal skin fold chamber model of cancer growth (Kemler et al–submitted) where again we observed cells in close proximity to highly infected foci that did not become infected for the duration of the experiment.

### Simulations of virus spread in 2D and 3D

**Fig 7:**
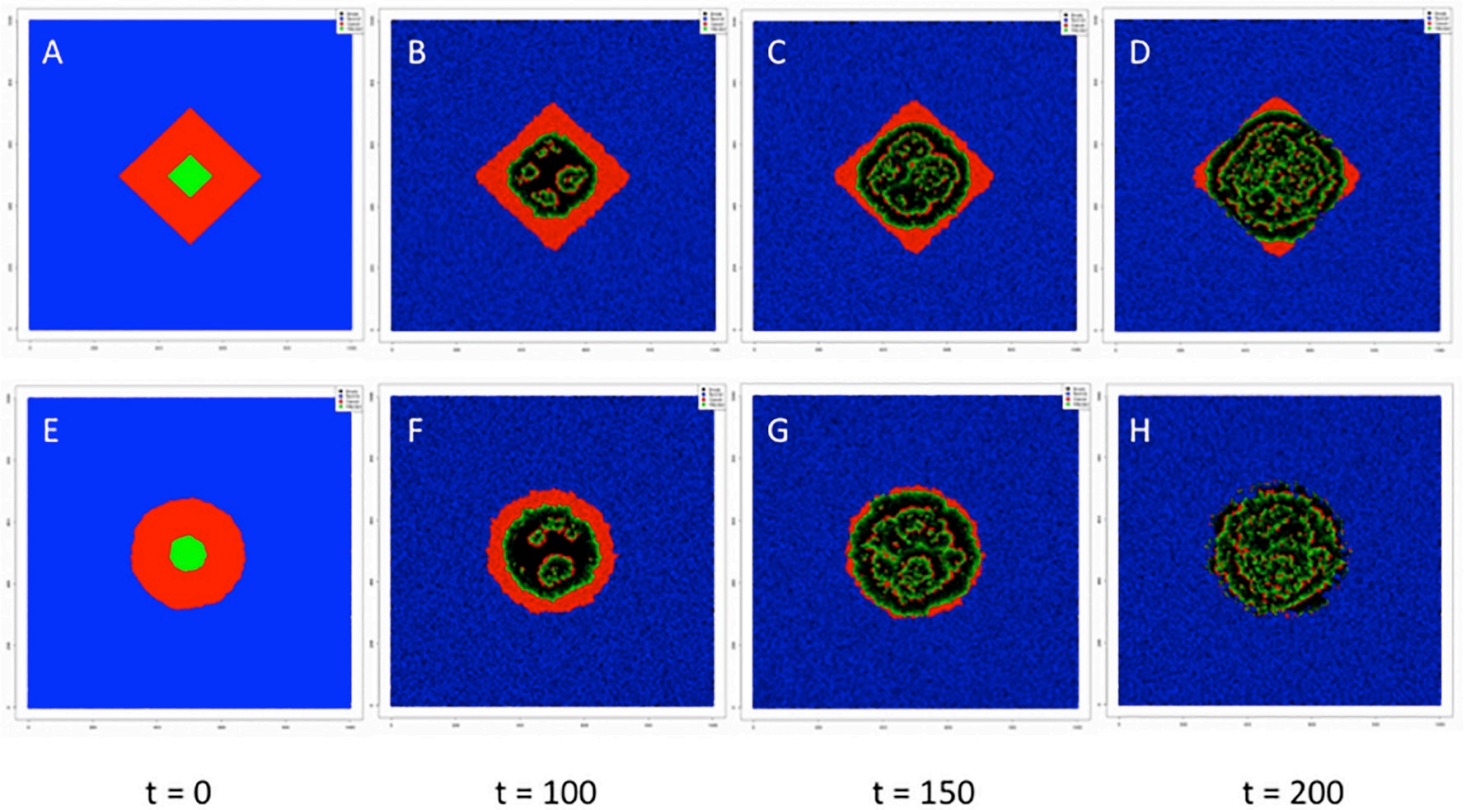
Two dimensional in silico simulations. Snapshots of the spread of an oncolytic virus in a 2D environment with a regular lattice architecture (A-D) having *n* = 4 nearest neighbors and with a Voronoi lattice (E–H) with *n* = 6 nearest neighbors. In both scenarios, there is one major focus of infection as in the equivalent in vitro scenario. Blue represents normal cells, red represents tumor cells, green represents infected tumor cells and black represents empty space.

**Fig 8:**
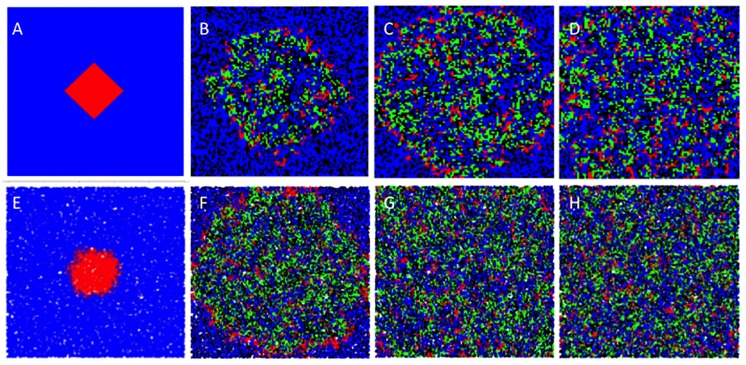
In silico simulation of virotherapy in 3 dimensions. Serial snapshots of the spread of an oncolytic virus in a 3D environment with a regular lattice architecture (A-D) having *n* = 6 nearest neighbors and with a Voronoi lattice (E–H) with *n* = 16 nearest neighbors. In both scenarios, multiple foci of infection are present as in the equivalent in vitro scenario. Even a single focus of infection ultimately results in independent foci of virus propagation. This may provide a means of escape for the tumor. Blue represents normal cells, red represents tumor cells, green represents infected tumor cells and black represents empty space. The yellow represents the equilibrium when only tumor cells and virus coexist.

We utilized these observations to perform *in silico* simulations of cell dynamics either in the 2D plane or in 3 dimensions each under two scenarios: growth on a regular lattice or growth on a Voronoi lattice. We studied the dynamics across a wide range of parameter estimates (Figs 7 and 8). All four networks studied had 1 × 10^6^ nodes with the 2D networks having a dimension of 1000 × 1000 while the 3D networks had 100 × 100 × 100 dimensions. At the start of each simulation, 90% of the nodes were occupied by normal cells, 9% were occupied by cancer cells and the initial viral inoculum infects 1% of the tumor cell population. If these simulations were allowed to run on an infinitely large and complete network (appropriately defined), the simulations would be stochastically identical to the mean field equations (see Methods).

Starting with simulations in 2D, for the set of parameters chosen, simulations led to equilibria with the three cell populations present. The time to reach an equilibrium in the 2D regular lattice architecture was ~6000 time units (average number of neighbors: 4), while in the 2D Voronoi lattice (average number of neighbors: 6), the time to equilibrium was 5000 time units. In the case of 3D simulations, the time to equilibrium on the regular lattice (average number of neighbors: 6) was 250 time units, while in the case of the 3D Voronoi lattice (average number of neighbors: 16), the average time to equilibrium was 150 time units. Therefore, the main determinant of the speed to reach equilibrium is the dimensionality of the network more than the number of neighbors, although the latter is also important. In Figs 7 and 8, we illustrate specific examples of such simulations in 2D (Fig 7) and 3D (Fig 8). In parallel, we also determined the results of the mean field solutions given by the mathematical model. It is clear that the mean field solution overestimates the effect of therapy with a larger population of infected tumor cells at equilibrium both in the 2D and 3D simulations. The mean field solution also overestimates the speed at which equilibrium is reached. Spread of the virus in 3D leads to a larger fraction of tumor cells infected at equilibrium compared to the 2D scenario but overall the tumor cell population is larger at equilibrium in the 3D network and illustrates the difficulty of controlling the 3D tumor compared to the tumor cells growing in vitro. There are also striking differences in the pattern of infection in 2D versus 3D that again illustrates the role of connectivity between cells.

### Simulations and equilibrium analysis

**Fig 9:**
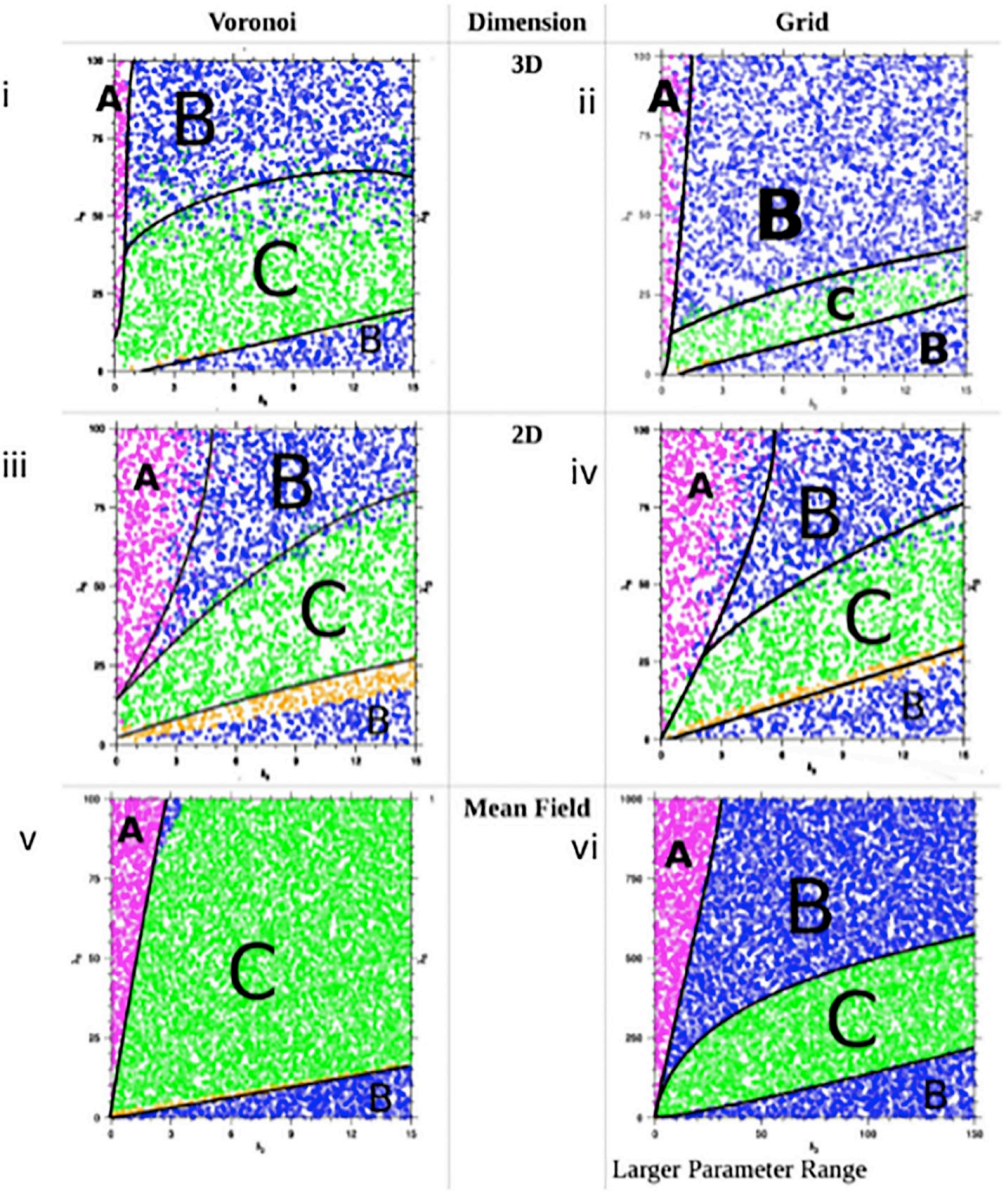
Critical properties of an oncolytic virus. The equilibrium results of simulations performed in 3D and 2D both in a Voronoi lattice (i and iii) and in a regular lattice (ii and iv) are plotted with the variables being the speed of virus replication (λ) and infected cell death (δ). The mean field solution (v) is also presented for comparison. (vi) represents the mean field solution over a wider range of parameters). Red (A) represents tumor eradication, Blue (B) represents the equilibrium with the virus and normal cell elimination and green (C) represents the co-existence of all three populations.

There are five outcomes of tumor virotherapy regardless of the model and number of dimensions considered. (i) The tumor population will go extinct and the virus infected tumor population will soon follow, leading to permanent cure of the tumor. (ii) The virus infected cell population goes extinct and the result will be the eventual takeover of the simulation space by the tumor cells since they grow faster than normal cells. This will mean that therapy has failed. (iii) The three populations of cells co-exist and have a (non zero) stable size. This will imply partial success of therapy. (iv) Normal cells are eliminated and at equilibrium only tumor cells and infected tumor cells coexist. (v) All populations die out. We do not consider the last scenario in our simulations. We were mainly interested in the range of virus specific parameters that maximize the chance of tumor elimination. Using data from prior work on in vivo tumor control with the same virus [8–11], we fixed the replication and natural death rates of normal and cancer cells and varied the parameters for virus replication and virus induced cell death rates across a wide range of values (*λ*_3_: 0 − 100; *δ*_3_: 0 − 15). A total of 14,000 simulations were performed with each simulation continuing until either the tumor cell population was eliminated or 1000 days had passed, whichever came first. We report the cumulative results of these simulations in Fig 9. As can be seen, the results are qualitatively different. The mean field solution predicts that most of the time, the 3 population equilibrium is the most likely outcome. In 2 dimensions, the parameter range where cure of the tumor is possible is wider compared both to the mean field estimate and the 3D simulations. Moreover, in 2D there is very little difference in output between the regular grid lattice and the Voronoi lattice likely due to the fact that the number of nearest neighbors is similar (4 versus 6 respectively). However, the probability of a cure is less for a Voronoi type network in 3D compared to a regular lattice, although the Voronoi lattice increases the chances for the co-existence of all three populations with less chance of the tumor taking (failed therapy) over compared to the 3D regular lattice. This is likely due to the higher number of neighbors that each cell possesses which increases the chance for infection. All simulations agree that the ideal virus should replicate rapidly (high *λ*) but kill cells slowly (low *δ*). Indeed, the model shows that there is a wider tolerance for replication rates and less so for the death rates of infected cells. Tumor eradication is more likely on a 2D surface compared to a 3D object for any set of parameters even though in 3D the number of cell neighbors is higher and equilibrium is reached faster, implying faster dynamics of virus spread. This is compatible with our in vitro observations and illustrates some of the intrinsic barriers to virus spread imposed by a 3D architecture versus a surface.

## Discussion

Tumor therapy with replication-competent viruses is an exciting novel approach to cancer therapy in which the target to be eliminated amplifies the agent responsible for its own death. Perhaps the only other member of this paradigm is cancer immunotherapy with chimeric antigen receptor T cells that are stimulated to replicate by engagement of cell surface antigens expressed by tumor cells. However, for successful tumor control with viruses, the latter have to establish foci of infection within the tumor, replicate to amplify the virus population and spread across the tumor. At the same time, the virus has to evade as much as possible the immune response that can neutralize the virus population or eliminate infected cells which would halt virus propagation. The outcome of such therapy is highly dependent on the dynamic interactions between the various populations of cells [5–7, 23–26]. However, as our results show, the outcome is also quite sensitive to the architecture of the tumor since there may be several barriers to the spread of the oncolytic virus. These barriers may be physical or chemical in nature [13, 27]. It has been argued that modeling with differential equations that provide a mean field approximation may be good enough to optimize therapy with these viruses and that such equations can capture well the dynamics without the need to consider space explicitly [26]. We have addressed the problems with this postulate in our work.

Initially we provided a detailed analysis of tumor cell growth and virus spread in vitro both in 2D and 3D coupled with an analysis of the rate of replication of cells as well as the number of neighboring cells in a given environment. Our modeling approach differs from other publications [17] since we used this data to generate realistic computational models of tumor cell growth and virus spread. Subsequently, we analyzed through simulations many potential scenarios that consider two critical virus parameters: its rate of replication and the rate at which it kills cells. These two parameters have been repeatedly shown to be important for the outcome of virotherapy [6, 8–10, 12, 13, 17, 18, 28]. The wide spectrum of viruses available for oncolytic therapy have different kinetics of spread or can be engineered to alter their kinetics of replication or cell killing [1]. Our in vitro studies show that the same virus will kill cells more slowly in a 3D environment compared to the 2D setting, despite the fact that in 3D the average number of cells in a neighborhood is higher. As a result, in 3D, for the same set of parameters, the probability of tumor elimination is lower compared to the mean field approximation and also less than in a 2D environment despite the system reaching equilibrium by at least an order of magnitude faster. This fits well with all in vitro studies where the virus is highly efficient in killing tumor cells growing in the 2D plane but less so in a 3D environment whether in vitro (spheroids) or in vivo as tumor xenografts [8–12, 29]. There are several possible explanations for the difference in outcome for tumor control in 2D versus 3D. While in 2D virtually all the tumor population is likely accessible to the virus that spreads at a fast rate, the same cannot be said for the 3D scenario where geometry not only makes some areas of the tumor quite distant from the infected foci but the virus also physically appears to spread at a slower rate even between adjacent cells. Moreover, one can envisage scenarios in 3D where a part of the tumor loses contact with the main tumor that is being infected. This will impose even greater spatial restrictions on the spread of the virus and reduces the probability of tumor control even further. However, in 3D the equilibrium is reached faster due to the higher number of contacts between cells.

It is not difficult to see why the mean field model will overestimate the effect of therapy, since this approach assumes the presence of a well-mixed population based on mass action kinetics, thus rendering tumor cells accessible to viruses at all times. However, in a 3D structure such as a tumor, not all cells are at the same risk of being infected due to their spatial proximity, or lack thereof, to infected foci. Any part of the tumor that loses contact with infected foci will result in tumor regrowth unless virus can diffuse and establish a new infection there–something that appears to be unlikely with the current scenarios [27]. Moreover, we have observed from in vivo studies that many cells in proximity to a highly infected focus never become infected and the size of infected foci can be quite variable (Kemler et al, submitted). The biological and physical bases for these observations require further analysis.

Our work complements that of Wodarz et al [7, 19] who studied spread of an adenovirus in a monolayer and showed the importance of local interactions on the spread of the virus. Their work expanded on the importance of initial conditions and the potential impact of an antiviral state due to interferon production. It is important to note that 293T cells used in their experiments are not derived from a tumor and so are likely to respond to interferon production. In contrast, we used cell lines that are derived from tumors that generally do not mount a robust immune response against measles virus. We also extended our in vitro studies and computational modeling to 3D where the number of neighbors and the spatial structures become more complex.

Our results show that the number of neighbors surrounding a cell can facilitate spread of the virus and leads to the three population equilibrium more often and more quickly (compare Voronoi network with grid lattice in Fig 9). However, in the presence of an immune response, we hypothesize that the outcome could be worse for a Voronoi type lattice compared to a regular lattice since the latter has a higher probability of a cure. The simulations also show that the mean field solution generally provides a more optimistic view of the outcome compared to the Voronoi type architecture that seems to exist in spheroids. However, the higher number of neighbors in a Voronoi network is associated with failure of therapy less often compared to a regular grid lattice at least in theory.

Our results highlight the need for spatially explicit modeling to accurately capture the dynamics of tumor virotherapy. We also show the problems that arise from the introduction of a third dimension into such a model–the probability of a cure decreases significantly when the virus is used in an attempt to cure a 3D tumor compared to cells in the 2D plane.

## Materials and models

### Cell lines

The human fibrosarcoma cell line HT1080 was obtained from ATCC and grown in Dulbecco’s modified Eagle’s medium (DMEM) supplemented with 10% fetal bovine serum (FBS) and maintained at 37°C with 5% CO_2_. 293T cells were maintained in DMEM with 10% FBS. The human cell line 293-3-46 was maintained in DMEM with 10% FBS and geneticin (1.2mg/ml) while Vero cells were maintained in DMEM with 5% FBS.

### Lentiviral vectors expressing fluorescent proteins

PCR products containing the yellow fluorescent protein (YFP), enhanced blue fluorescent protein (eBFP), and the tdTomato genes were generated using Roche Fast-Start High Fidelity PCR kit using pCAG-YFP, CFP, pCSCMV:tdTomato (Addgene) as template DNA. The primers (Forward: GGGATCCACGCCACCATGGTGAGCAAGGGCG and reverse: GAGGCGGCCGCAGTTTACTTGTACAGCTCGTCCATGCC) had restriction sites for *BamHI* and *NotI* to facilitate cloning in the lentiviral vector backbone [30]. PCR products were cloned into pHR`CMV-eGFP-SIN (a gift of Dr Y. Ikeda, Mayo Clinic, Rochester) after the excision of GFP via digestion with *BamHI* and *NotI*. The resulting constructs were verified by restriction digestion and sequencing. Lentiviral particles containing the respective reporter genes were generated via transfection of 293T cells with pMD.G, pΔCMV.8.91, and the plasmid encoding the vector genome with the fluorophore as previously described [30]. Vector containing supernatants were harvested after 72 hours, filtered (0.42μm) and used to transduce HT1080 cells. Cells expressing the reporter gene were sorted by flow cytometry and plated as single cells into 96 well plates in DMEM with 10% FBS and expanded as clones for further studies.

### Recombinant measles virus generation

In order to generate recombinant replication competent measles viruses expressing different fluorescent reporter genes, PCR products containing the fluorophores eBFP, YFP, and tdTomato were generated using pCAG template DNA (Addgene). PCR primers had the *MluI* and *AatII* restriction sites (underlined) in their flanking region to facilitate cloning (Forward: GACGCGTACGCCACCATG GTGAGCAAGGGCG and reverse: GAGACGTCAGTTTACTTGTACAGCTCGTC CATGCC. The PCR products were gel purified, digested and subsequently ligated into pCR2.1Topo, expanded in TOP10 cells (Invitrogen), and inserts excised with *MluI* and *AatII* digestion followed by ligation into p(+)MVeGFP(N) that was digested with the same enzymes to remove the eGFP gene. Viruses were rescued by transfection of 293-3-46 cells together with pEMC-La followed by overlay on Vero cells as previously described [4, 31]. Rescue of the recombinant viruses was inferred from the presence of syncytia and fluorophore detection under ultraviolet light. The recombinant viruses were expanded by infection of Vero cells. Cell associated viruses were freed by freeze thawing of the cells three times in liquid nitrogen followed by filtration. The viral titers (50% infectious virus dose, TCID_50_/ml) were determined using the Spearman and Karber method as previously described [4, 31]. All viruses were stored at -80°C until they were used.

### Tumor spheroid generation

HT1080 spheroids were grown on either Matrigel coated glass bottom 6 well plates or poly-HEMA coated round bottom plates to prevent cell attachment. For Matrigel coated wells, media, tips, and glass bottom plates were cooled at 4°C and 200μl Matrigel added. Matrigel was allowed to solidify for 15 minutes. HT1080 cells were washed 3 times with phosphate buffered saline (PBS) and dislodged by trypsin, counted and overlaid at various concentrations into 2ml DMEM with 10%FBS and 2% Matrigel. The media were freshly replaced every 3–4 days and cells were imaged with a multiphoton microscope (Olympus). Spheroids were infected using measles viruses encoding various fluorophores in 2 mL Opti-MEM for 2 hours at 37°C at an MOI of 1.0.

### In vitro imaging

HT1080-tdTomato cells were plated in 6 well plates and 24 hours later infected with MVeGFP at an MOI of 1.0 Tumor spheroids from the same cell line expressing eCFP were produced as above and infected with MV-tdTomato. Starting twenty-four hours post infection, the cells were imaged in Z stacks every 30 minutes over the course of a week using an Olympus multiphoton microscope. Digital images were captured for subsequent analysis.

### Pattern analysis

Average pixel area per cell: The average pixel area of a cell was determined by having two independent scientists counting the number of cells in a given field of view with a fixed color pixel threshold and correlated this with the phase contrast images of the cells. The total pixel area was divided by the number of cells to determine the average pixel area per cell. Serial images of in vitro cell growth and virus spread were digitally analyzed using the established cell area parameters and the output was converted back into number of cells.

Voronoi tessellation: Digital images from the in vitro experiments with cells growing in the 2D-plane or in 3D spheroids were analyzed using MatLab to generate Voronoi tessellation analysis of nearest neighbors in 2D and 3D.

### Mathematical modeling and data fitting

We developed a ‘mean field’ mathematical model for tumor growth and viral infection of tumor cells as follows:

$$\frac{du_{N}}{dt}=\lambda_{N}u_{N}(1-u_{N}-u_{C}-u_{V})-\delta_{N}u_{N}$$

$$\frac{du_{C}}{dt}=\lambda_{C}u_{C}(1-u_{N}-u_{C}-u_{V})-\delta_{C}u_{C}-\lambda_{V}u_{C}u_{V}$$

$$\frac{du_{V}}{dt}=-\delta_{V}u_{V}+\lambda_{V}u_{C}u_{V}$$

In the model, *u*_*i*_ represents the various cellular fractions with *N* representing normal cells, *C* cancer cells and *V* the infected cancer cells. *λ*_*i*_ represents the proliferation and *δ*_*i*_ the death rates of the respective populations. The model assumes mass action kinetics. The model was fitted to data from in vitro studies using the purpose built simplex induction hybrid (SIH) program [32] and the results of the fits displayed as a heat plot (see results). The goodness of fit was determined using the chi square method. The relative parameters for virus infected cells and tumor cells were used to inform the computer simulations.

### Computational model

The model we developed can run simulations of cell populations and infection both in 2 and 3 dimensions and population growth can be either on a lattice structure (regular) or a Voronoi network with a variable number of neighboring cells [18]. The input for the network is described by a list of adjacencies for each node obtained by analysis of the imaging obtained from in vitro experiments. The simulator itself has no dimensionality and therefore, it can run simulations in two, three or higher dimensions. The number of neighbors for each node and their location can vary as observed in vitro. Routines embedded in the program enable modification of the state of the network to simulate the addition of the virus that will spread within the cell population.

Each node in the network is occupied at most by one cell. Three types of cells are possible: normal cells, cancer cells and infected cancer cells. A node without a cell is empty. When a normal or cancer cell proliferates, the new cell generated has to occupy a neighboring empty node while infected cells can target only nodes occupied by a cancer cell since the virus is tumor cell specific [33] and the virus spreads from cell to cell [18, 21, 31]. Each cell type has a node type that it considers as a replication target. Empty nodes are proliferation targets for normal and cancer cells while a cancer cell node is a proliferation target for infected cells. The growth and death rates of normal and cancer cells as well as infected cells can be varied as well as the time when the virus is administered. The virus infection parameters can also be specified. Event arrivals follow a Poisson process with the time to the next event being exponentially distributed. Furthermore, the model assumes that cell proliferation and death are independent events and do not depend on the prior state of the network.

To initiate the infection, the program determines the coordinates of all cancer cells and identifies the cancer cell that is closest to the center. The simulator has 4 infection routines that can be used to specific which individual nodes are infected at the start of the simulation. (i) *Random* selects cells at random within the tumor population until the pre-specified fraction of cells are infected. (ii) *Center* selects the cancer cell closest to the center of the tumor and this cell is infected followed by its neighbors and neighbors’ neighbors are infected until the required fraction of infected cells is reached. (iii) *Multinode* selects the tumor cells closest to the center for infection and the infection spreads from this focus by generating a line in a random direction that passes through the infected node. Nodes along the line are visited and if they are cancer cells infected with a determined probability. The process continues until normal cells are reached which cannot be infected. (iv) *Perimeter* determines the center of the tumor and the distance of all nodes from the center. Cancer cells occupying the nodes furthest from the center are infected until the pre-specified fraction of cancer cells are infected.

**Fig 10:**
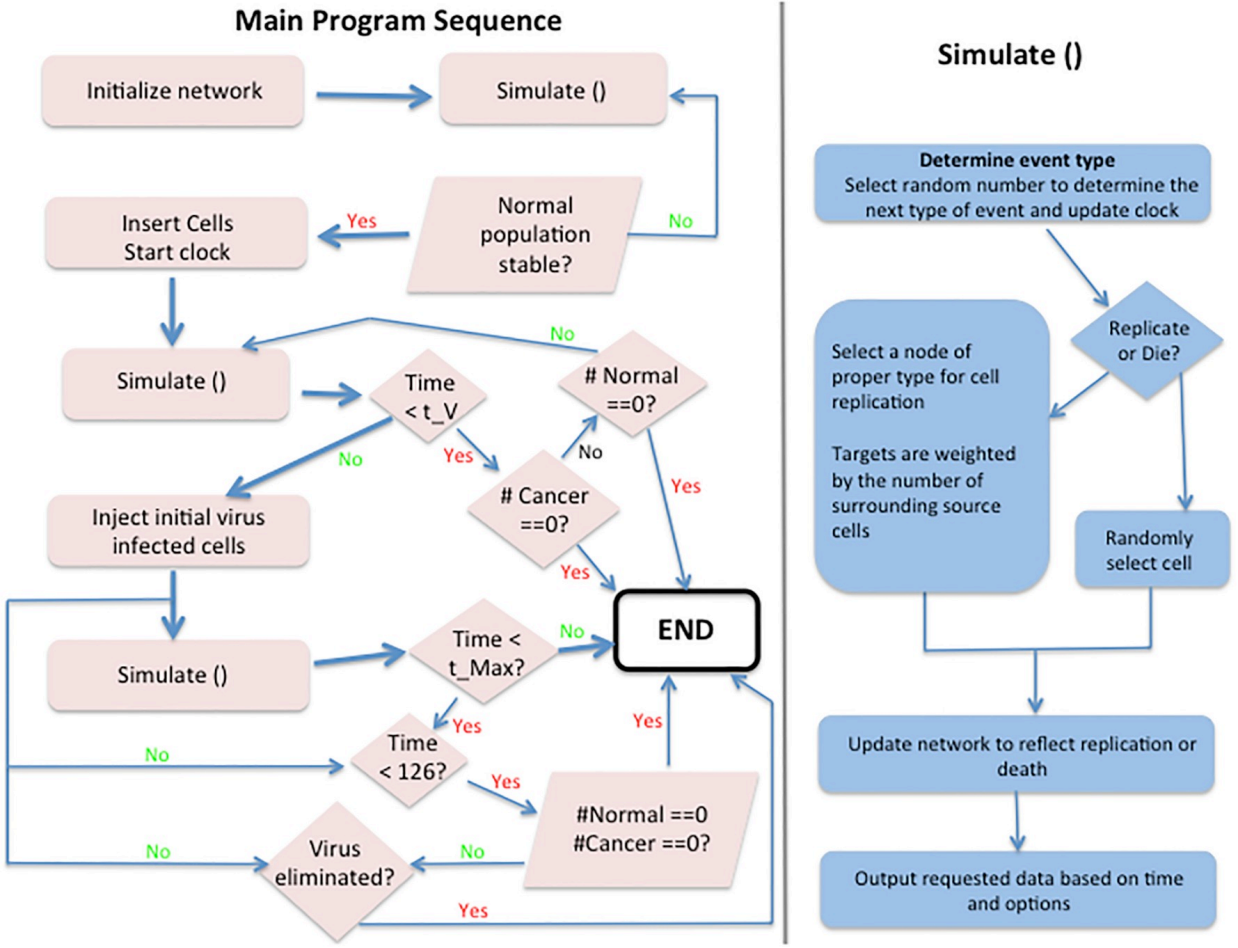
Schematic representation of the computer code used for simulations. The code requires input for population size, distribution of near neighbors and replication and death rates for normal, cancer and infected cancer cells. The virus can be added to the simulation at any arbitrary time.

The dynamics of normal and uninfected cancer cells continue with the same parameters once the infection is introduced. A simulation starts by reading a set of network files that specify the network structure, node locations and cell types (optional). If no cell type is included, normal cells are assumed. The program has an optional equalization period until the population stabilizes. Cancer cells are then introduced and the simulation allowed to run until the virus is introduced. The simulation is continued until either the cancer or virus population goes extinct or a pre-specified time is reached. The percentage of infected cells at the time of virus administration is an input variable. Initial infection is assumed to occur very rapidly. In all simulations, time is defined by the rate of replication of cells (the generation time). Fig 10 presents a schematic of the process. A copy of the code is available at http://hdl.handle.net/11299/174710. A total of 7,000 runs were performed for each set of spatially explicit simulations (e.g. 2D grid, 2D Voronoi) starting with similar initial conditions but varying the parameters related to the rate of virus spread (*λ*_3_ = 0 − 100) and virus induced cell death rate (*δ*_3_ = 0 − 15).
